# Superinfection of an Obstructive Appendiceal Mucocele: A Case Report

**DOI:** 10.7759/cureus.23974

**Published:** 2022-04-09

**Authors:** Christofis Charalambous, Thalis Charalambous, Aristotelis Nikitaras, Prokopis Christodoulou

**Affiliations:** 1 Radiology Department, Alexandra General Hospital, Athens, GRC; 2 Department of Neuroinflammation, University College London (UCL) Institute of Neurology, Queen Square Multiple Sclerosis (MS) Center, London, GBR; 3 First Surgical Department, General Hospital Asklepieion Voulas, Athens, GRC

**Keywords:** abdominal pain, ct (computed tomography) imaging, appendix, superinfection, appendicular mucocele

## Abstract

Appendiceal mucocele is an uncommon entity that may arise due to benign or malignant processes. The radiologic exploration of this entity is necessary for diagnosis, and its imaging manifestations vary, with some findings being more common than others. More specifically, the radiological findings of a superinfected mucocele are rare, with few reports in the literature. Herein we present the case of a 68-year-old male patient with a superinfected appendiceal obstructive mucocele caused by a fecalith, which was diagnosed by abdominal CT evaluation.

## Introduction

Mucocele of the appendix is a rare clinical entity that may have either benign or malignant behavior regarding its type. Simple obstructive mucocele may be caused by fecalith or stricture or epithelial hyperplasia, and its incidence is up to 29% [1]. On the other hand, malignant appendiceal mucoceles may arise from a mucinous cystadenoma in a range of 31%-34% and mucinous adenocarcinoma in a range up to 5% [1]. A major concern in mucinous lesions of the appendix is their preoperative diagnosis by radiology exploration as the surgical approach is different between benign and malignant mucoceles, and the surgeon must be aware of the intact mucocele’s removal to prevent pseudomyxoma peritonei [2]. Superinfected mucocele is a radiological term that refers to the presence of intraluminal air bubbles or air-fluid levels in the mucocele [3]. Bennet et al. found out that the presence of superinfection was 8.3% in a retrospective evaluation of CT features of appendiceal mucocele [2].

## Case presentation

**Figure 1:**
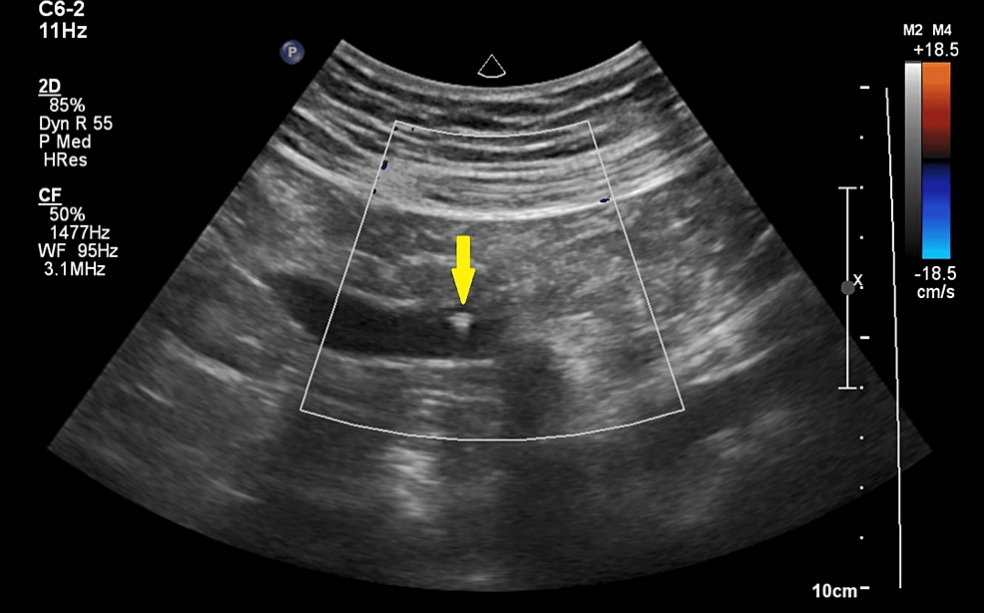
Image of the appendix during the abdominal ultrasound the yellow arrow indicates the appendicolith.

A 68-year-old male was presented to the Emergency Department of our hospital due to mild pain in the right iliac and peri-umbilical region without any other symptoms such as nausea and anorexia. The patient reported that the pain started 10 hours ago, in the same region, without any other reflection of it. His past medical history was unremarkable. On arrival, his vital signs were normal, and the clinical examination revealed slight tenderness at the right iliac region, a negative McBurney sign, and normal bowel sounds. Full blood examinations and electrocardiogram (ECG) showed slight elevation of the white blood cells (WBC) and C-reactive protein (CRP). ECG was normal. The patient was then referred to the Radiology Department for an abdominal ultrasound which revealed a tubular hypoechoic cystic mass with no peripheral or internal vascularity at the right lower quadrant. An appendicolith with posterior acoustic shadowing was observed inside the cystic mass. No peri-appendix edema or wall-thickening was detected (Figure 1).

**Figure 2:**
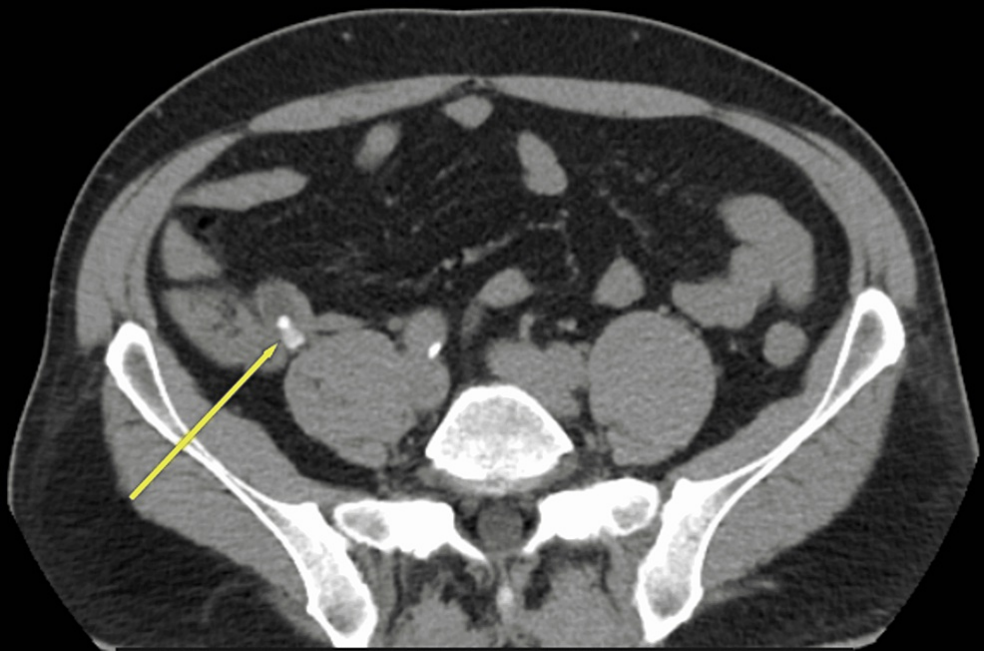
Axial view of abdominal CT, the yellow arrow indicates the obstruction of the appendix by appendicolith.

**Figure 3:**
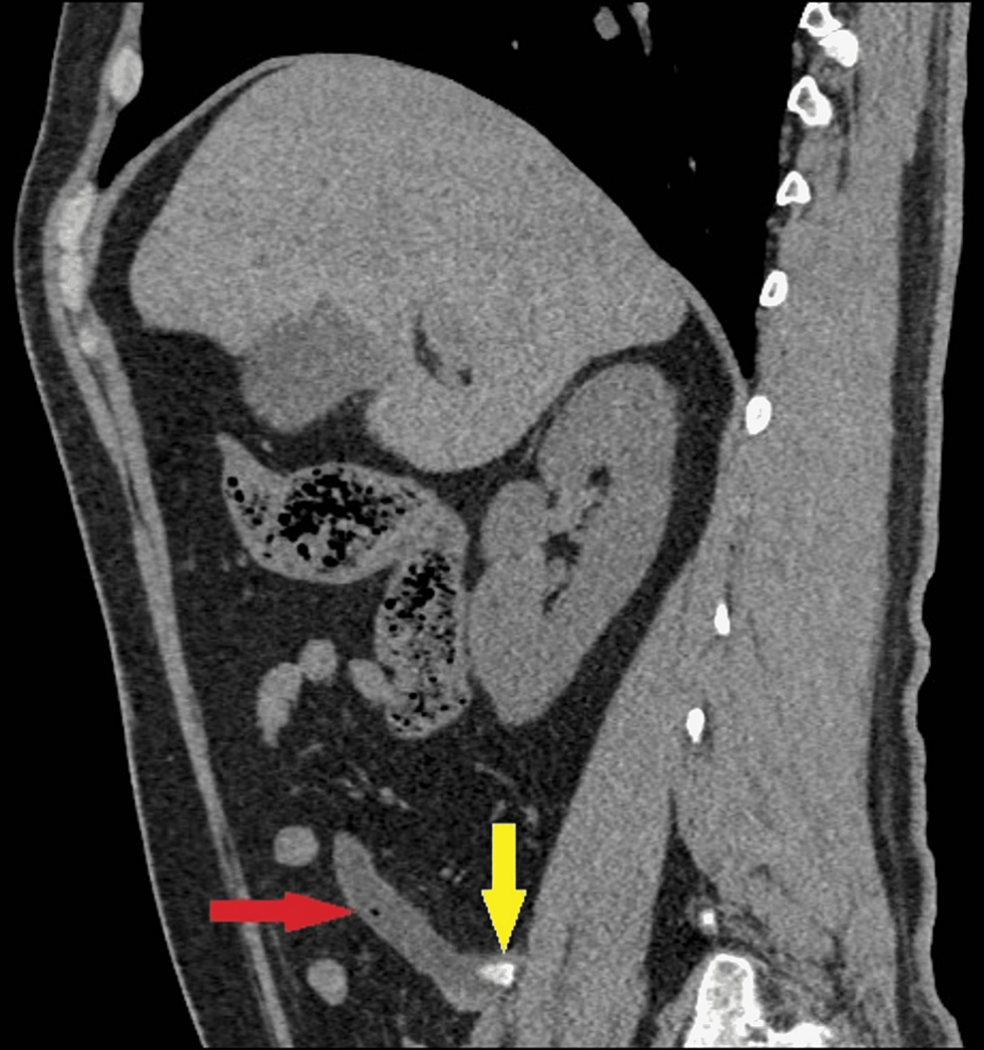
Sagittal view of abdominal CT showing dilated appendix, the red arrow indicates the superinfection of appendiceal mucocele, and the yellow arrow indicates the appendicolith.

An abdominal CT scan was then performed for further evaluation without administration of gastrografin or intravenous (IV) contrast material due to a history of allergic reaction to a prior CT exam. The abdominal CT scan revealed a well-circumscribed, low attenuation tubular mass that was continuous with the base of the caecum. At the initial segment of the appendiceal mucocele, an appendicolith was observed, which causes mucus retention due to obstruction (Figure 2). Mild fat stranding and a small number of intraluminal bubbles of air were seen, a sign of a superinfection (Figure 3). The maximum diameter of the finding was 1.51 cm. The patient was then returned to the Emergency Department, where the surgeons suggested to the patient admission to the surgical ward and the performance of an open appendectomy. The patient denied it as he preferred to refer elsewhere.

## Discussion

Appendiceal mucocele (AM) is a rare clinical entity caused by benign or malignant appendix lesions, and its incidence is estimated up to 0.7% [4]. The definition of AM refers to a dilated appendix full of mucus due to benign or malignant obstruction [4,5]. It can occur mostly in females between 50 to 70 years old [6]. Mucinous lesions of the appendix are classified into two categories according to the Peritoneal Surface Oncology Group International (PSOGI), which are non-neoplastic mucinous lesions such as simple mucocele or retention cysts due to inflammatory or obstructive processes and neoplastic mucinous lesions which include mucinous appendiceal neoplasms, mucinous adenocarcinomas, and serrated appendicular polyps [7,8]. AM can be asymptomatic, picked up incidentally in radiology examinations or intraoperatively, or symptomatic mimicking acute appendicitis [9]. Cestino et al. presented three cases with different clinical presentations wherein one patient presented with acute appendicitis, the second patient was an incidental finding in an abdominal CT scan, and the third patient was incidental intraoperative finding while the patient led to the operation room with the suspicion of intestinal intussusception after abdominal CT scan [10].

The most common non-neoplastic cause is a simple mucus retention cyst due to luminal obstruction. Simple mucus retention cysts usually measure less than 2 cm, whereas neoplastic mucoceles tend to be larger at presentation [4]. In ultrasound, a mucocele appears like an ovoid cystic mass in the right lower quadrant, and the internal echogenicity varies [11]. Internal concentric echogenic layers that give the appendix an “onion-skin” appearance and acoustic shadowing due to mural calcifications are features highly suggestive of a mucocele [11]. Mural calcifications are seen in less than 50% of the cases [3,12].

On CT, the mucocele appears as a dilated appendix filled with homogeneous low attenuation material. Features like mural nodularity and irregular wall thickening are associated with malignant mucoceles due to adenocarcinoma [11]. The mural calcifications are seen in less than 50% of the cases on CT too. An infected mucocele will develop intraluminal gas or air-fluid levels, wall thickening, and fat stranding, although these findings can overlap with those associated with a primary tumor [10,13]. On MRI, mucocele manifests characteristics of the simple fluid, but the signal intensity may vary depending on the specific protein content [11]. The identification of an appendiceal mucocele should prompt a search for extraluminal mucin in the periappendice space, peritoneal cavity, or the surface of abdominal and pelvic organs, including the ovaries and bowel [14].

The management of appendiceal mucocele is exclusively surgical; however, the type of surgery depends on whether the mucocele is benign or malignant [15]. Among open and laparoscopic surgery, the option of the open approach is favorable as it has higher possibilities to prevent rupture of the malignant mucocele, which may lead to mucinous spread into the peritoneal cavity and cause a condition called pseudomyxoma peritonei which is associated with poor prognosis and further management may be needed with cytoreductive surgery and hyperthermic intraoperative peritoneal chemotherapy [4,15,16]. Two types of surgical resection are appropriate for AM: appendectomy or right hemicolectomy [15,17]. Right hemicolectomy is suggested if there is the involvement of ileocolic or appendiceal lymph nodes or if the histopathology report reveals a non-mucinous neoplasm, or in cases that are necessary to perform right hemicolectomy to achieve complete resection of the primary tumour or complete cytoreduction [17].

## Conclusions

Appendiceal mucocele includes a diverse clinical entity regarding its behavior, cause, diagnosis, and treatment. We highlight the necessity of correct evaluation of radiological findings to determine the right treatment plan for the patients. Moreover, independently of the presence of benign or malignant mucocele is important; the intact removal of the specimen while rupture may lead to pseudomyxoma peritonei.
